# Supplementary material for: A Pilot Study on Efficacy of Lipid Bubbles for Theranostics in Dogs with Tumors
Source: Cancers (Basel). 2020 Aug 26;12(9):2423. doi: 10.3390/cancers12092423 (PMC7564957; doi:10.3390/cancers12092423)
Supplement: Supplementary file 1 [file cancers-12-02423-s001.pdf]

cancers

MDPI

Article

A Pilot Study on Efficacy of Lipid Bubbles for Theranostics in Dogs with Tumors

Inoru Yokoe [1], Yusuke Murahata [1], Kazuki Harada [1], Yuji Sunden [1], Daiki Omata [2], Johan Unga [2], Ryo Suzuki [2], Kazuo Maruyama [3], Yoshiharu Okamoto [1] and Tomohiro Osaki [1,*]

1 Joint Department of Veterinary Clinical Medicine, Faculty of Agriculture, Tottori University, 4-101 Koyama-Minami, Tottori 680-8553, Japan; inoru.y@gmail.com (I.Y.); ymurahata@muses.tottori-u.ac.jp (Y.M.); k-harada@tottori-u.ac.jp (K.H.); sunden@tottori-u.ac.jp (Y.S.); yokamoto@muses.tottori-u.ac.jp (Y.O.)
2 Laboratory of Drug and Gene Delivery Research, Faculty of Pharma-Science, Teikyo University, 2-11-1 Kaga, Itabashi-ku, Tokyo 173-8605, Japan; omata@pharm.teikyo-u.ac.jp (D.O.); johan.unga@gmail.com (J.U.); r-suzuki@pharm.teikyo-u.ac.jp (R.S.)
3 Laboratory of Theranostics, Faculty of Pharma-Science, Teikyo University, 2-11-1 Kaga, Itabashi-ku, Tokyo 173-8605, Japan; maruyama@pharm.teikyo-u.ac.jp (K.M.)
* Correspondence: tosaki@muses.tottori-u.ac.jp; Tel.: +81-857-31-5434; Fax: +81-857-31-5434

Supplementary Material

Anti-tumor effects of LBs combined with US and liposomal doxorubicin

Table S1. Summary of the three dogs included in the clinical study of the combination treatment of LBs, US, and Doxil.

9	Breed	Age (y)	Sex	Weight (kg)	Tumor type	Tumor site	Simultaneous therapy
1	Papillon	14	Spayed female	2.5	Hemangiopericytoma	Perianal	None
2	Toy Poodle	7	Female	3.8	Ceruminous gland adenocarcinoma	Left ear	None
3	Welsh Corgi	10	Female	10.1	Thyroid carcinoma (suspected)	Thyroid	Toceranib

Table S2. Summary of the clinical course and treatment of case No. 1.

Day	Events	Tumor size (cm³)	Doxil dose (mg/m²)	LBs dose (mL)	US output setting Frequency (MHz)	Power Intensity (W/cm²)	Duty Cycle (%)	Time (min)
**0**	**Inclusion 1st treatment**	**178.0**	**16**	0.2	1	2	50	30
9	2nd treatment	175.7	16	0.2	1	2	50	20
23	Poor general condition Treatment postponed	85.6	n/a[1]	n/a[1]	n/a[1]	n/a[1]	n/a[1]	n/a[1]
28	Slight improvement of general condition Treatment postponed	77.8	n/a[1]	n/a[1]	n/a[1]	n/a[1]	n/a[1]	n/a[1]
52	Death	n/a[1]	n/a[1]	n/a[1]	n/a[1]	n/a[1]	n/a[1]	n/a[1]

[1] n/a, not assessed.

Table S3. Summary of the clinical course and treatment of case No. 2.

Day	Events	Tumor size (cm³)	Doxil dose (mg/m²)	LBs dose (mL)	US output setting Frequency (MHz)	Power Intensity (W/cm²)	Duty Cycle (%)	Time (min)
**0**	**Inclusion**	**4.4**	**16**	0.2	1	2	50	15

	**CT examination** **1st treatment**				1	2	100	15
7	2nd treatment	3.7	16	0.2	1	2	50	40
29	CT examination 3rd treatment	3.7	8	0.4	1	2	50	40
43	4th treatment	2.7	8	0.4	1	2	50	30
57	5th treatment	2.9	12.5	0.2	1	2	50	30
71	6th treatment	2.4	14.2	0.4	1	2	50	30
78	Inflammation of the lesion Treatment postponed	n/a[1]	n/a[1]	n/a[1]	n/a[1]	n/a[1]	n/a[1]	n/a[1]
113	Poor general condition Treatment postponed	n/a[1]	n/a[1]	n/a[1]	n/a[1]	n/a[1]	n/a[1]	n/a[1]
127	Death	n/a[1]	n/a[1]	n/a[1]	n/a[1]	n/a[1]	n/a[1]	n/a[1]

[1] n/a, not assessed.

Table S4. Summary of the clinical course and treatment of case No. 3.

**Day**	**Events**	**Tumor size (cm^3)**	**Doxil dose (mg/m^2)**	LBs dose (mL)	US output setting Frequency (MHz)	Power Intensity (W/cm^2)	Duty Cycle (%)	Time (min)
**0**	**Inclusion** **CT examination** **1st treatment**	**41.0**	**15**	0.2	1	2	50	15
15	2nd treatment	28.3	15	0.3	1	2	50	15
36	3rd treatment	38.2	15	0.3	1	2	50	15
58	CT examination 4th treatment	16.6	15	0.3	1	2	50	15
82	Poor general condition Treatment postponed	18.1	n/a[1]	n/a[1]	n/a[1]	n/a[1]	n/a[1]	n/a[1]
83	Poor general condition The last follow-up	n/a[1]	n/a[1]	n/a[1]	n/a[1]	n/a[1]	n/a[1]	n/a[1]

[1] n/a, not assessed.
